# Effects of olanzapine on anhedonia in schizophrenia: mediated by complement factor H

**DOI:** 10.3389/fpsyt.2023.1146714

**Published:** 2023-07-13

**Authors:** Yi Zhang, Wei Tang, Weiping Wang, Feikang Xu, Weihong Lu, Chen Zhang

**Affiliations:** ^1^Schizophrenia Program, Shanghai Mental Health Center, Shanghai Jiao Tong University School of Medicine, Shanghai, China; ^2^Department of Psychiatry, The Affiliated Kangning Hospital of Wenzhou Medical University, Zhejiang, China; ^3^Department of Psychiatry, Jinhua Second Hospital, Jinhua, Zhejiang, China; ^4^Shanghai Key Laboratory of Psychotic Disorders, Shanghai Mental Health Center, Shanghai Jiao Tong University School of Medicine, Shanghai, China

**Keywords:** anhedonia, schizophrenia, complement factor H, olanzapine, Snaith-Hamilton Pleasure Scale

## Abstract

**Background:**

Anhedonia is a trans-diagnostic symptom in schizophrenia and MDD. Our recent work indicated that increased plasma level of complement factor H (CFH) is associated with anhedonia in major depressive disorder. This study hypothesized that CFH is likely to be a biomarker of anhedonia in schizophrenia.

**Methods:**

A 12-week prospective study is performed to observe the effects of olanzapine on anhedonia and CFH. We used the Chinese version of Snaith-Hamilton Pleasure Scale (SHAPS) to evaluate anhedonic phenotype in patients with schizophrenia. Plasma levels of C-reactive protein (CRP), C3, C4 and CFH were measured.

**Results:**

Of the recruited 152 samples, patients with anhedonia were found in 99/152 (65.13%). Patients with anhedonia had notably higher PANSS negative subscores, SHAPS total score and higher level of plasma CFH than those without anhedonia (*P*s<0.05). Stepwise multivariate linear regression analysis showed that increasing level of plasma CFH was a risk factor for SHAPS total score (*β* = 0.18, *p* = 0.03). Of the 99 patients with anhedonia, 74 completed the 12-week follow-up. We observed significantly reduced scores of PANSS, SHAPS and decreased plasma CFH level, when the patients completed this study. The change of SHAPS total score is positively correlated with the level of CFH decrease (*p* = 0.02).

**Conclusion:**

Our results implied that plasma CFH levels may be a biomarker for anhedonia in schizophrenia, and the effect of olanzapine on treating anhedonia is through decreasing plasma CFH levels.

## 1. Introduction

Schizophrenia is a disabling and chronic mental illness, with a prevalence of 1% population in world (1). Although the understanding of its pathophysiology remains unknown, epidemiological data have indicated a possible correlation between immune system and schizophrenia (2). There is also accumulating evidence showing the shared features between schizophrenia and certain autoimmune diseases (3). The recent genetic studies have consistently reported an association of schizophrenia and the major histocompatibility complex, supporting the theory that immune dysfunction may enhance risk for this illness (4). Collectively, the abovementioned findings imply immune involvement in the pathophysiology of schizophrenia.

It is known that complement system plays an important role in innate and adaptive immune functions (5, 6), in which C3 and C4 yield critical physiological functions, and Complement factor H (CFH) regulates the activation of the alternative pathway (7, 8). In our previous studies, we did not detect any association of C3 and C4 with schizophrenia (9, 10). However, we have recently found that the gene encoding CFH (*CFH*) influences negative symptoms in schizophrenia (11). Anhedonia is considered as a core negative symptom of schizophrenia (12, 13). Our most recent work has shown that increased plasma level of CFH is associated with anhedonia in major depressive disorder (MDD) (14). Given the evidence that anhedonia is a trans-diagnostic symptom in schizophrenia and MDD (15), there is no study to detect the relationship of CFH with anhedonia in schizophrenia.

In the past years, second-generation antipsychotics (SGAs) have been widely prescribed to treat schizophrenia. Olanzapine is a commonly and well used SGAs in clinical practice. Our previous studies showed that olanzapine yields improvement of negative symptoms in patients with schizophrenia through anti-inflammatory effects (16, 17). Literature documented that 16-week olanzapine monotherapy significantly improves anhedonia in first-episode schizophrenia patients (18). The effect of olanzapine on anhedonia in chronic mild stress (CMS)-exposed rats is also observed in preclinical studies (19). As such, olanzapine may have a therapeutic effect on anhedonia, whereas its underlying mechanism is still unclear. We hypothesized that CFH may be a potential biomarker of anhedonia in schizophrenia and the effect of olanzapine in alleviating anhedonia may be through modulating the expression of CFH.

Thereby, we performed a 2-stage study to validate our hypothesis. A genome-wide association study has identified that peripheral levels of C3 and C4 are influenced by CFH in Han Chinese (20). As such, we first recruited drug-naïve patients with first-episode schizophrenia to test whether plasma levels of complement system correlate with anhedonia in patients with schizophrenia. Subsequently, we carried out a 12-week clinical observation to investigate whether olanzapine alleviates anhedonia through modulating complement system.

## 2. Methods

### 2.1. Subjects

The inclusion criteria of this study have been reported previously (17, 21). The samples were recruited from four mental hospitals in Eastern China, including Jinhua Second Hospital, Shanghai Mental Health Center, Wenzhou Kangning Hospital and Wuxi Mental Health Center. All recruited samples received olanzapine monotherapy.

### 2.2. Clinical evaluation

The psychotic symptoms was assessed using the Positive and Negative Syndrome Scale (PANSS). The anhedonic symptom was assessed using the Chinese version of Snaith-Hamilton Pleasure Scale (SHAPS), referring to our previous work (14). This 14-item self-report questionnaire has a good reliability and validity in Chinese culture (22). Higher SHAPS scores manifest a higher level of anhedonic symptom, and individuals with anhedonic symptom were defined as total SHAPS scores>5.

### 2.3. Plasma levels of inflammatory parameters analysis

Plasma levels of C3, C4, CFH and CRP were all measured with enzyme-linked immunosorbent assay (ELISA) kits (RayBiotech, Norcross, GA). The experimental participants were blinded to all the clinical data.

### 2.4. Statistical analysis

SPSS 20.0 was used for quantitative and qualitative variables. Independent sample t-test was used to test the variables that conformed to normal distribution, and Mann–Whitney U-test were used to compare the variables that did not conform to normal distribution between groups. A stepwise regression model based on the forced entry method was used to detect the correlation of plasma levels of inflammatory parameters with psychotic symptoms and anhedonia in schizophrenia patients. PANSS and SHAPS total score was selected as dependent variable, and inflammatory parameters were assessed as independent variables. Treatment efficacy of olanzapine from baseline to endpoint was assessed by Wilcoxon signed rank test. Spearman’s correlation analysis was used to calculate the correlation of inflammatory parameters with changes of PANSS and SHAPS total score. *p* < 0.05 was considered statistically significant.

## 3. Results

**Figure 1:**
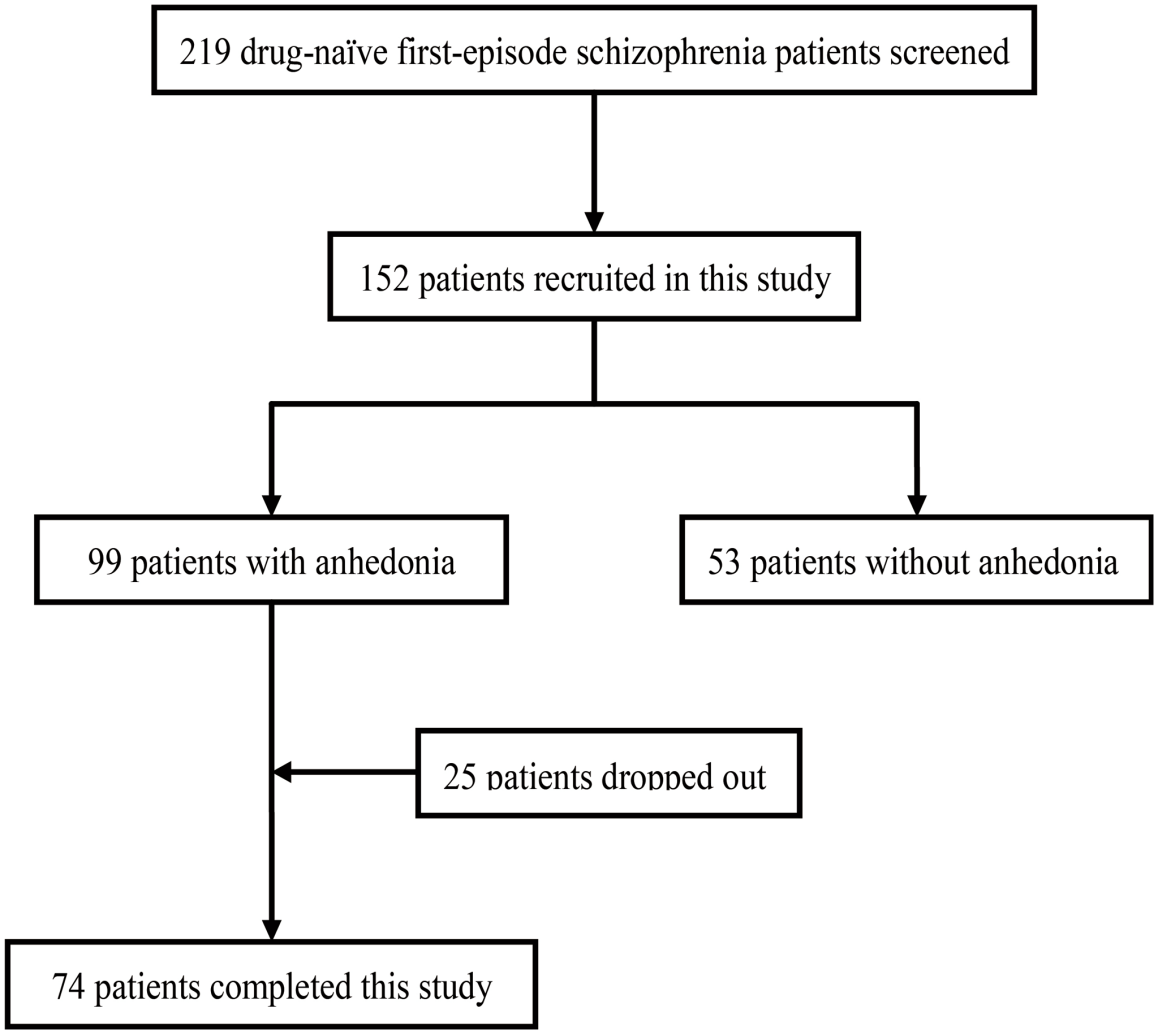
Study flow chart.

The flow chart of this study was presented in Figure 1. For the cross-sectional study, there were 219 first-episode drug-naïve patients with schizophrenia screened. One hundred and fifty two patients were recruited and received baseline assessments. Of these, patients with anhedonia were found in 99/152 (65.13%). Clinical characteristics and inflammatory parameters of the patients with or without anhedonia were presented in Table 1. There was no difference between with or without anhedonia groups in terms of age, sex, duration of illness. Patients with anhedonia had notably higher PANSS negative subscores, SHAPS total score and higher level of plasma CFH than those without anhedonia (*P*s<0.05). Stepwise multivariate linear regression analysis using SHAPS total score as the dependent variable and CRP, C3, C4 and CFH as the independent variables revealed that increasing level of plasma CFH was the independent risk factor for SHAPS total score (*β* = 0.18, *p* = 0.03, shown in Table 2). As shown in Supplementary Table S1, we found that CRP and C3 was the independent risk factor for PANSS positive score (*β* = −0.08, *p* = 0.02 and *β* = 45.82, *p* = 0.01, respectively).

**Table 1:** Clinical characteristics and inflammatory parameters of patients with or without anhedonia at baseline.

Characteristic	With anhedonia (*n* = 99)	Without anhedonia (*n* = 53)	Normality (*P*)	Statistics (χ^2^)	*P*
Sex (M/F)	62/37	27/26		1.94	0.17
				*t*/*Z*	*P*
Age (years)	27.51 ± 3.98	27.23 ± 5.65	0.20	0.32	0.75
Duration of illness (months)	7.79 ± 4.49	6.81 ± 3.78	<0.01	−2.88	<0.01
PANSS					*P* ^a^
Positive subscore	22.78 ± 4.88	24.02 ± 3.20	0.11	2.64	0.11
Negative subscore	27.44 ± 2.86	23.94 ± 3.14	<0.01	−8.36	<0.01
General psychopathology	32.95 ± 3.39	33.49 ± 2.79	0.04	−1.52	0.13
Total score	83.17 ± 6.09	81.45 ± 4.96	0.07	2.45	0.12
SHAPS	10.07 ± 2.25	2.85 ± 1.06	<0.01	−10.19	<0.01
CRP	6.67 ± 1.44	6.28 ± 1.35	0.57	1.62	0.11
C3	2700.84 ± 680.79	2476.99 ± 946.94	0.93	1.52	0.13
C4	1785.19 ± 873.25	1619.30 ± 1006.98	0.04	−1.63	0.10
CFH	426.13 ± 62.29	401.02 ± 93.49	0.06	−2.76	0.02

Data presented as $\bar{x}$ ± s.

a The *p* values were adjusted for age, gender and duration of illness.

**Table 2:** Multivariate linear regression analysis with SHAPS total score as the dependent variable in patient with schizophrenia.

	*B*	SE	*β*	*t*	*P*
CRP	0.22	0.23	0.08	0.96	0.34
C3	0.13	0.16	0.11	1.39	0.17
C4	0.08	0.06	0.15	1.83	0.07
CFH	0.21	0.14	0.18	2.20	0.03

B, unstandardized coefficients; SE, standard error; β, standardized coefficients.

**Figure 2:**
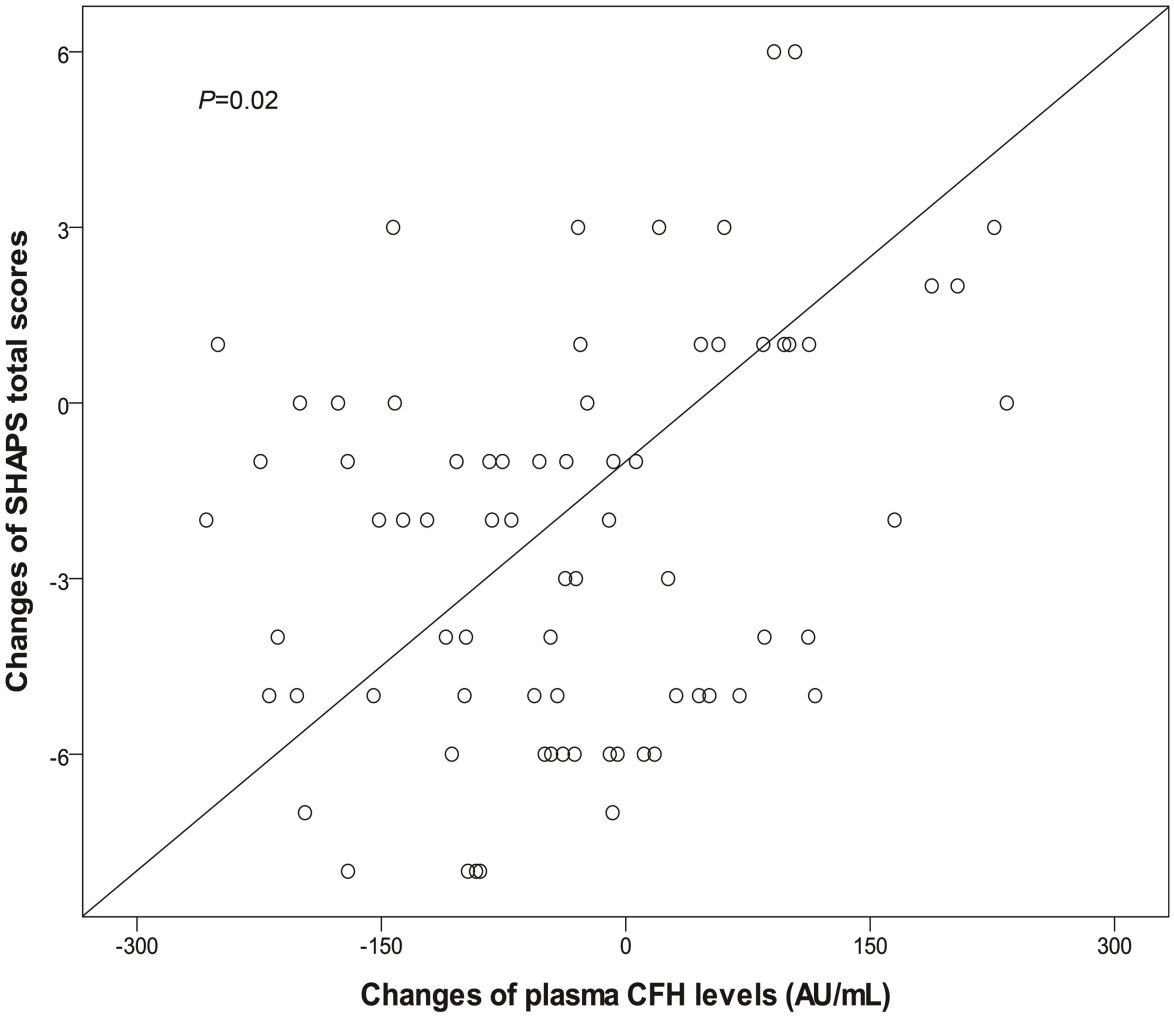
Significant correlation between changes of plasma CFH levels and SHAPS total scores after 12-week olanzapine monotherapy. CFH, complement factor H; SHAPS, Snaith-Hamilton Pleasure Scale.

For the prospective observation study, 99 patients were enrolled in treatment. Twenty-five patients dropped out for various reasons. All data reported here are based on the remaining 74 patients who completed this study. The primary endpoint was the changes of PANSS, SHAPS and plasma CFH level from baseline to 12^th^ week. As shown in Table 3, we observed significantly reduced scores of PANSS, SHAPS and decreased plasma CFH level, when the patients completed this study. Subsequently, we conducted the Spearman’s correlation analysis to detect whether the changes of PANSS and SHAPS total score is correlated with decreased level of CFH. Figure 2 showed that the change of SHAPS total score is positively associated with the decreased level of CFH (*p* = 0.02). We also observed a marginally significant association between PANSS positive subscore and CFH (*p* = 0.04), as shown in Supplementary Figure S1.

**Table 3:** Comparison of clinical characteristics and plasma CFH level between baseline and endpoint (12th week) in group with anhedonia.

	Baseline	Endpoint	Change	*Z*	*P*
PANSS positive subscores	21.09 ± 4.08	15.23 ± 2.06	−7.73 ± 2.83	−6.96	<0.01
PANSS negative subscores	27.86 ± 2.61	18.14 ± 4.05	−9.72 ± 4.99	−7.81	<0.01
PANSS general subscores	33.78 ± 2.51	23.72 ± 3.31	−10.07 ± 4.18	−7.43	<0.01
PANSS total score	82.74 ± 5.57	57.08 ± 5.58	−27.53 ± 7.23	−7.47	<0.01
SHAPS	10.00 ± 2.27	7.65 ± 2.16	−2.35 ± 3.39	−4.90	<0.01
CFH (AU/mL)	435.01 ± 75.32	401.71 ± 95.65	−33.29 ± 115.28	−2.40	0.017

## 4. Discussion

In clinical practice, anhedonia appears trans-diagnostically in schizophrenia and MDD. According to the DSM-5 criteria, anhedonia is a hallmark of MDD as well as a core negative symptom of schizophrenia (23). Our recent study has documented that the complement function is implicated with anhedonia in patients with MDD (14). In this study, we firstly aimed to identify the role of complement system in anhedonic symptom in schizophrenia. As such, we tested the inflammatory marker CRP, and complement system including C3, C4 and CFH between schizophrenia patients with or without anhedonia. Our results showed that plasma level of CFH is significantly higher in schizophrenia patients with anhedonia than those without anhedonia. Further stepwise multivariate linear regression analysis showed that increasing level of plasma CFH was the independent risk factor for SHAPS total score. These results implied that enhanced plasma level of CFH is implicated with anhedonia in schizophrenia. Our results also showed that CRP and C3 may be the independent risk factor positive symptoms in schizophrenia. This is line with previous studies (24).

We previously found that schizophrenia patients have higher levels of *CFH* expression in brain than healthy subjects, and *CFH* is associated with severity of negative symptoms in schizophrenia (11). It is known that abnormalities of *CFH* can impair the normal homeostasis of complement system (25). Our previous study pointed out that increased level of *CFH* mRNA is positively correlated with severity of Alzheimer’s disease (AD), and *CFH* results in structural change of the atrophy rate and entorhinal cortex during AD progression (26). The most recent neuroimaging study has showed that decreased entorhinal thickness is correlated with clinical phenotypes including anhedonia in patients with MDD (27). A preclinical work observed that neonatal lesions of the entorhinal cortex decreased motivation during operant behavior, reflecting a state of anhedonia in rats (28). These findings suggested that increasing CFH level may result in anhedonia through influencing structural and function of entorhinal cortex. Accordingly, CFH may be a potential biomarker for anhedonia in patients with schizophrenia.

As such, we secondly conducted a 12-week prospective longitudinal study to observe the effect of olanzapine on psychotic symptoms and anhedonia in schizophrenia. Our results showed that 12-week olanzapine improved positive and negative symptoms and anhedonia for the treatment of first-episode, drug-naïve schizophrenia patients. This is in agreement with previous studies (17, 18, 29). Preclinical work also indicated that olanzapine has a protective effect against anhedonia in a rodent model of depression (30). Our previous study found that olanzapine exerts neurotrophic effect and promotes neuroplasticity and synapse formation through modulating inflammatory pathways (17, 31, 32). Given the potential role of CFH in anhedonia, we subsequently detected the effect of olanzapine on CFH and anhedonia in patients with schizophrenia. Our results showed that olanzapine attenuated plasma level of CFH, and the changes of CFH were positively correlated with the changes of SHAPS total score and PANSS positive subscore. This suggested that olanzapine may alleviate anhedonia and positive symptoms in patients with schizophrenia through decreasing CFH level. Animal experiment showed that anhedonia is derived from decreasing strength of hippocampus-nucleus accumbens synapses and impaired long-term potential (LTP) (33). It is known that complement proteins in brain tag synapses to mediate synaptic development and promote synaptic plasticity (34, 35). CFH is a complement control protein and displays an important role in mediating synaptogenesis (36). A proteome study showed a mixture of increased and decreased phosphorylation of peripheral complement proteins in response to the 6-week olanzapine treatment in patients with schizophrenia (37). Our previous work indicated that that olanzapine rescues impaired LTP in hippocampus induced by MK-801 (31). There is evidence showing that olanzapine increases synapse formation and neurogenesis through modulating the levels of inflammatory and neurotrophic factors (38). As such, we assumed that the effect of olanzapine on alleviating anhedonia may depend on strengthening synaptic plasticity by controlling complement pathway. However, the underlying mechanism is still required for further investigations.

This study had some limitations. First, it was a multi-center study, and the sample size was limited. Thus, generalization of the results beyond the population should be made cautiously. Second, this study is the absence of placebo control. Finally, causal relationships could not be determined from this cross-sectional study whereas we reported a weak significance between CFH with anhedonia in patients with schizophrenia. As such, further investigations are required to validate our findings in this study.

In summary, this 2-stage study was conducted to identify the biomarker for anhedonia in patients with schizophrenia. Our evidence is suggestive that plasma CFH levels could be suggested as a potential biomarker for anhedonia in schizophrenia, and the effect of olanzapine on treating anhedonia is through decreasing plasma CFH levels.

## Data availability statement

The raw data supporting the conclusions of this article will be made available by the authors, without undue reservation.

## Ethics statement

The studies involving human participants were reviewed and approved by Shanghai Mental Health Center Ethics Committee, Wuxi Mental Health Center Ethics Committee, Jinhua Second Hospital Ethics Committee, and Wenzhou Kangning Hospital Ethics Committee. The patients/participants provided their written informed consent to participate in this study.

## Author contributions

YZ and CZ contributed to the overall design of the study and wrote the manuscript. YZ, WT, WW, FX, and WL got involved in sample collection. YZ, WT, and WL undertook the statistical analysis and interpretation of data. All authors contributed to the article and approved the submitted version.

## Funding

This work was supported by the National Key Research and Development Program of China (2018YFC1314302), the National Natural Science Foundation of China (81471358 and 81771450), the Shanghai Science and Technology Commission Foundation (19411969300 and 21Y11905700), and Medical and Public Health Program for Social Development in Wuxi (WX18IIAN032). The funding bodies had no role in the design of the study and collection, analysis, interpretation of data, and in the writing of the manuscript.

## Conflict of interest

The authors declare that the research was conducted in the absence of any commercial or financial relationships that could be construed as a potential conflict of interest.

## Publisher’s note

All claims expressed in this article are solely those of the authors and do not necessarily represent those of their affiliated organizations, or those of the publisher, the editors and the reviewers. Any product that may be evaluated in this article, or claim that may be made by its manufacturer, is not guaranteed or endorsed by the publisher.
