# Supplementary material for: Effects of olanzapine on anhedonia in schizophrenia: mediated by complement factor H
Source: Front Psychiatry. 2023 Jul 13;14:1146714. doi: 10.3389/fpsyt.2023.1146714 (PMC10372489; doi:10.3389/fpsyt.2023.1146714)

**Supplementary Table S1 Multivariate linear regression analysis with inflammatory parameters as the dependent variable and PANSS as the independent variables in patient with schizophrenia**

| CRP | B | SE | *β* | *t* | *P* |
| --- | --- | --- | --- | --- | --- |
| Positive subscore | -0.08 | 0.03 | -0.20 | -2.37 | 0.02 |
| Negative subscore | -0.04 | 0.40 | -0.09 | -1.05 | 0.29 |
| General psychopathology | 0.03 | 0.04 | 0.05 | 0.65 | 0.52 |
| Total score | -0.04 | 0.02 | -0.12 | -1.53 | 0.13 |
| C3 |  |  |  |  |  |
| Positive subscore | 45.82 | 17.71 | 0.21 | 2.59 | 0.01 |
| Negative subscore | 35.08 | 21.32 | 0.14 | 1.65 | 0.10 |
| General psychopathology | 37.76 | 24.19 | 0.13 | 1.56 | 0.12 |
| Total score | 40.33 | 12.43 | 0.26 | 3.25 | 0.01 |
| C4 |  |  |  |  |  |
| Positive subscore | 0.94 | 21.44 | 0.01 | 0.04 | 0.97 |
| Negative subscore | 12.16 | 25.81 | 0.04 | 0.47 | 0.64 |
| General psychopathology | -0.95 | 29.28 | -0.01 | -0.03 | 0.97 |
| Total score | 3.65 | 15.04 | 0.02 | 0.24 | -0.81 |
| CFH |  |  |  |  |  |
| Positive subscore | 0.84 | 1.96 | 0.04 | 0.43 | 0.67 |
| Negative subscore | -1.03 | 2.36 | -0.04 | -0.44 | 0.66 |
| General psychopathology | -4.57 | 2.68 | -0.14 | -1.71 | 0.09 |
| Total score | -1.29 | 1.39 | -0.08 | -0.93 | 0.35 |

Note: B: unstandardized coefficients; SE: standard error; *β*: standardized coefficients

**Supplementary Figure S1 Correlation between changes of PANSS and CFH from baseline and endpoint (12^th^ week) in group with anhedonia.** A. Correlation of PANSS positive subscores with CFH; B. Correlation of PANSS negative subscores with CFH; C. Correlation of PANSS general subscores with CFH; D. Correlation of PANSS total scores with CFH


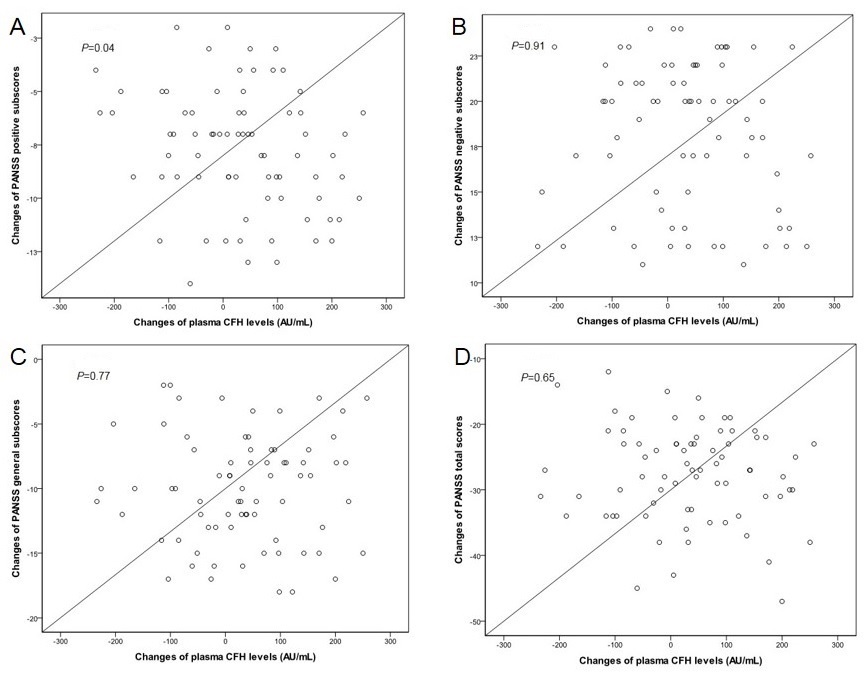

Supplement: Supplementary file 1 [file Data_Sheet_1.docx]
